# “Technology has allowed us to do a lot more but it’s not necessarily the panacea for everybody”: Family physician perspectives on virtual care during the COVID-19 pandemic and beyond

**DOI:** 10.1371/journal.pone.0296768

**Published:** 2024-02-29

**Authors:** Lindsay Hedden, Sarah Spencer, Maria Mathews, Emily Gard Marshall, Julia Lukewich, Shabnam Asghari, Paul Gill, Rita K. McCracken, Crystal Vaughan, Eric Wong, Richard Buote, Leslie Meredith, Lauren Moritz, Dana Ryan, Gordon Schacter

**Affiliations:** 1 Faculty of Health Sciences, Simon Fraser University, Burnaby, British Columbia, Canada; 2 Department of Family Medicine, Schulich School of Medicine and Dentistry, Western University, London, Ontario, Canada; 3 Primary Care Research Unit, Department of Family Medicine, Dalhousie University, Halifax, Nova Scotia, Canada; 4 Faculty of Nursing, Memorial University, St. John’s, Newfoundland and Labrador, Canada; 5 Family Medicine, Faculty of Medicine, Memorial University, St John’s, Newfoundland and Labrador, Canada; 6 Department of Family Practice, Faculty of Medicine, University of British Columbia, Vancouver, British Columbia, Canada; Access Alliance Multicultural Health and Community Services: Access Alliance, CANADA

## Abstract

**Introduction:**

Early in the COVID-19 pandemic, Canadian primary care practices rapidly adapted to provide care virtually. Most family physicians lacked prior training or expertise with virtual care. In the absence of formal guidance, they made individual decisions about in-person versus remote care based on clinical judgement, their longitudinal relationships with patients, and personal risk assessments. Our objective was to explore Canadian family physicians’ perspectives on the strengths and limitations of virtual care implementation for their patient populations during the COVID-19 pandemic and implications for the integration of virtual care into broader primary care practice.

**Methods:**

We conducted semi-structured qualitative interviews with family physicians working in four Canadian jurisdictions (Vancouver Coastal health region, British Columbia; Southwestern Ontario; the province of Nova Scotia; and Eastern Health region, Newfoundland and Labrador). We analyzed interview data using a structured applied thematic approach.

**Results:**

We interviewed 68 family physicians and identified four distinct themes during our analysis related to experiences with and perspectives on virtual care: (1) changes in access to primary care; (2) quality and efficacy of care provided virtually; (3) patient and provider comfort with virtual modalities; and (4) necessary supports for virtual care moving forward.

**Conclusions:**

The move to virtual care enhanced access to care for select patients and was helpful for family physicians to better manage their panels. However, virtual care also created access challenges for some patients (e.g., people who are underhoused or living in areas without good phone or internet access) and for some types of care (e.g., care that required access to medical devices). Family physicians are optimistic about the ongoing integration of virtual care into broader primary care delivery, but guidance, regulations, and infrastructure investments are needed to ensure equitable access and to maximize quality of care.

## Introduction

Early in the COVID-19 pandemic, Canadian primary care practices rapidly adapted to provide care virtually (synchronous visits via telephone or video interfaces) to minimize exposure and transmission risks for themselves and their patients [1, 2]. Prior to 2020, most Canadian jurisdictions lacked or had only limited remuneration for virtual care. As a result, family physicians’ (FPs’) virtual offerings were limited [3, 4] and Canada lagged behind other Organization for Economic Cooperation and Development countries in its uptake of virtual care despite demand from patients [5–7]. At the outset of the pandemic, for most Canadian FPs, virtual care was a new delivery modality for which they lacked prior training or expertise [2]. By mid-2020, in response to COVID-19, virtual care accounted for more than 70% of total primary care consultations in Ontario [1, 8]. Between March 2020 and June 2021, the proportion of family physician consultations provided virtually ranged between 27% and 57% in Manitoba, Saskatchewan, and British Columbia [9]. By March 2022, 49% of Canadians report that they were offered a virtual visit along with non-virtual modalities [10].

Research conducted prior to the COVID-19 pandemic suggested that approximately 66% of primary care visits require an in-person service [11]. However, there is sufficient evidence to support the effectiveness of virtual care for certain services, including monitoring, communication and counselling patients with select chronic illnesses, and the provision of psychotherapy as part of behavioural health interventions [12]. Despite this evidence, there remains limited formalized guidance to support physicians in their decision-making on which visits can be conducted virtually, and which require an in-person appointment [13–15]. Early in the pandemic, much of the available guidance was limited to instructions from medical professional organisations and governments, instructing physicians to use virtual consultations “wherever possible” [16–19], with little to no indication of which care warranted an in-person appointment.

This lack of clinical guidance, meant that physicians made individual decisions about in-person versus virtual care based on their clinical judgement, longitudinal relationships with their patients, and personal risk assessments [2]. Concerns about privacy, safety and quality of care also impacted decisions about whether care should be provided in-person or virtually [20–23]. This resulted in varied use and implementation of virtual care during the pandemic, and informed physicians’ perceptions about how virtual care should be integrated into regular primary care practice.

Our objective was to explore Canadian FPs’ perspectives on the strengths and limitations of virtual care approaches–namely, telephone and video visits–for their patient populations during the COVID-19 pandemic, implications for primary care planning for future public health emergencies, and the integration of virtual care into broader primary care practice. This work builds on a previous companion paper that captures the experiences of FPs with the transition to virtual care during the pandemic [2].

## Methods

### Setting and design

This analysis has been conducted as part of a larger study, *Pandemic Planning for Primary Care* [24], for which we conducted qualitative interviews with family physicians in four regions in Canada: Vancouver Coastal health region in British Columbia [25], Ontario Health West [26], Nova Scotia [27], and Eastern Health in Newfoundland and Labrador (which has since been consolidated into a single Provincial Health Authority [28–30]). These regions were purposively selected due to variability in their primary care structures, practice models and remuneration policies, and variations in their COVID-19 public health responses.

### Participants, recruitment and data collection

We conducted semi-structured interviews with FPs who were clinically active in one of the four study regions between 1 October 2020 and 15 June 2021. We excluded students and post-graduate medical residents, as well as physicians who did not provide clinical care (e.g., who worked solely in academic or administrative roles) and those who did not hold an active license. We recruited participants using social media, outreach through privileging lists and practice directories, and snowballing (where permitted), and we followed a maximum variation approach to capture different career stages (i.e., early, mid, late career), models of practice (e.g., independent solo/group practice, community health centre, hospitalist, long term care) and remuneration (e.g., fee-for-service, alternative payment plan), genders, and community demographics (e.g., rural, urban, mixed) [31].

Interviews were 45–60 minutes in length and conducted either by phone or Zoom (Zoom Video Communications), based on participant preference, and followed a semi-structured interview guide (S1 File) that we developed with reference to the COVID-19 chronologies for each region. Participants were asked to comment on the roles they played (or were asked to play) during different phases of the pandemic and the barriers and facilitators they encountered in fulfilling those roles. Interviews were recorded, transcribed verbatim, and verified by interviewers. We continued with recruitment until we had sufficient data to facilitate rigorous analysis and no new themes were emerging (i.e., data saturation) [32, 33].

### Analysis

As pragmatists, we analyzed data using an inductive thematic analysis [34], within a broader qualitative descriptive approach [35]. Two team members in each of the four study regions reviewed a subset of interview transcripts and interviewer field notes and developed an initial coding framework. The broader research team then met to consolidate the region-specific frameworks into a single harmonized template that included coding criteria and descriptions for themes and subthemes. We refined the codes from broad and descriptive to analytic through iterative content analysis. Regional researchers then conducted full analysis of all transcripts and field notes using the harmonized template within NVivo V.12 (QSR International). Any coding disagreements between team members were resolved through discussion and consensus.

### Rigour and reflexivity

We developed our interview guides iteratively, and pre-tested them through consultation with physician team members and with reference to our evidence-informed, region-specific COVID-19 chronologies. We relied on experienced interviewers and conducted member checking during interviews [36, 37]. Member checking involved interviewers seeking confirmation or correction from participants as to their understanding of individuals’ reflections and experiences during their interview. We kept detailed records of interviews, field notes, and coding templates, as well as any coding disagreements and resolutions, and we encouraged self-reflection among all members of the research team [38]. This virtual care-focused analysis is led by a researcher with expertise in the expansion of virtual primary care in Canada, supported by active physicians and policy experts. Data collection was conducted by experienced qualitative interviewers. Our results are reported following the Standards for Reporting Qualitative Research (SRQR) [39].

All participants provided written informed consent, recognizing that their participation was voluntary. Data has been de-identified and is presented using participant codes.

## Results

We conducted interviews with 68 physicians across the four study regions (Table 1). All participants spoke about the transition to virtual care and its impact on themselves and their patients. Sixty percent (n = 41) of participants identified as women, and 68% (n = 46) reported receiving compensation under an alternative (i.e., not fee-for-service) payment model. Thirty percent (n = 20) worked in rural areas and 74% (n = 49) had hospital privileges. Key themes arising from these interviews included: (1) changes in access to primary care; (2) quality and efficacy of care provided virtually; (3) patient and provider comfort with virtual modalities; and (4) necessary supports for virtual care moving forward.

**Table 1:** Participant characteristics [n (%)].

	British Columbia	Ontario	Nova Scotia	Newfoundland & Labrador	Total
	N = 15	N = 20	N = 21	N = 12	N = 68
**Gender** ^**a**^					
Men	4 (26.7)	10 (50.0)	9 (42.9)	4 (33.3)	27 (39.7)
Women	11 (73.3)	10 (50.0)	12 (57.1)	8 (66.7)	41 (60.3)
**Remuneration Model**					
Fee-for-Service	6 (40.0)	4 (20.0)	7 (33.3)	5 (41.7)	22 (32.4)
Alternative Payment Plan^b^	9 (60.0)	16 (80.0)	14 (66.7)	7 (58.3)	46 (67.7)
**Hospital Privileges**					
No	3 (20.0)	5 (25.0)	6 (28.6)	5 (41.7)	19 (27.9)
Yes	12 (80.0)	15 (75.0)	15 (71.4)	7 (58.3)	49 (72.1)
**Community Size** ^**c**^					
Rural	0 (0)	9 (45.0)	8 (38.1)	3 (25.0)	20 (29.4)
Small Urban	0 (0)	1 (5.0)	0 (0.0)	0 (0.0)	1 (1.5)
Urban	15 (100.0)	8 (40.0)	13 (61.9)	8 (66.7)	44 (64.7)
Mix	0 (0)	2 (10.0)	0 (0.0)	1 (8.3)	3 (4.4)
**Years in Practice (mean)**	16.9 (SD = 8.2)	18.7 (SD = 9.8)	15.4 (SD = 10.0)	16.3 (SD = 11.1)	16.9 (SD = 9.8)

a Gender was asked as an open-ended question [40].

b Alternative payment plan includes all funding models outside of traditional and enhanced fee-for-service.

c Rural <10,000 population, Small urban = 10,000–99,999 population, Urban >1,000,000 population. Mix denotes where participants work in more than one community size.

### Changes in access to primary care

#### Expanded access options

Participants believed that the introduction of virtual care had the ability to provide a sense of security and utility during the pandemic, allowing some FPs to continue seeing their patients at a time when the risk of contracting COVID-19 by providing in-person care was high and access to personal protective equipment was limited. The balance of virtual to in-person interactions *“really ebbed and flowed”* [Participant S] as participants became more familiar with virtual modalities, and with both the level of COVID-19 circulating in the community and patient comfort levels:

*There’s been some evidence of community spread [of COVID-19] at various times*. *And so*, *when that happened*, *we would tend to really restrict in-person access to patients*. *And patients also were concerned*, *and rightly so*, *about being out and about in public*. [Participant S]

Beyond the context of a public health emergency, however, participants identified how virtual care had impacted patients’ experiences of primary care access. For some FPs, virtual care has minimized wait times for appointments: *“I went from a practice where patients were routinely waiting anywhere between two and four weeks to see me*, *to a practice where they’d get a call back within a week*, *or within a day if it was acute”* [Participant L]. Other participants in more rural and remote settings indicated how, with the introduction of virtual care, they would be able to continue providing access during bad weather when previously they would have cancelled or closed their clinic:

*Up here*, *we often will miss 2–3 clinics per winter because of snowfall and road conditions*. *So [virtual care] would be great for that—okay*, *we’re not going to be able to go to [Town] today to run the clinic*, *we’ll just switch to [videoconference platform] and we’ll see everybody over the internet*. [Participant R]

Participants also noted how virtual care could reduce barriers that impeded access to primary care, such as the need to take time away from work, arrange childcare, or deal with transportation, *“empower[ing] a lot of people to access their health care more”* [Participant P]. Consequently, some FPs whom we interviewed reported seeing more of their younger, healthier patients for routine and preventative care:

*I was getting many more touchpoints with younger*, *relatively healthy patients because they could contact virtually*, *right*? *[…] It would be very rare that I would have a young guy in his 40s come and say*, *“Hey*, *I’m feeling great but my dad died of a heart attack*, *and I need to talk to you about how I’m going to keep my cholesterol down and stay healthy*.*” Right*? *Because why would you ever take a day off work to do that*? [Participant L]

Participants also noted how virtual care made it easier to access certain patients with social anxiety and/or complex care needs. For some participants, virtual care had particular utility for seeing patients with mental health challenges on a more consistent basis: *“I have been able to provide more regular care to [patients with mental health challenges] because I wasn’t also negotiating with them […] the social anxiety of needing to get onto the bus”* [Participant F]. Others noted how virtual modalities facilitated more frequent contact with patients with chronic illnesses:

*Pre-COVID*, *I would see my really complex patients once a month*. *But during COVID*, *I would talk to them every two weeks or sometimes every one week–[virtual care] allowed me to micromanage a little bit more than I would if they had to come in person*. *[…] Like I can’t make them take time off*. *But I can call them on their cell phone at their lunch break at work and say*, *hey*, *is that medication working*? *How do you feel your anxiety is*? *Do you want to go up on the dose*? *I would never do that in person*. [Participant U]

#### Equity concerns

While most of the FPs in the study found that virtual care provided their patients with improved access, they also recognized the barriers that virtual care can pose for certain populations. This was particularly true for participants working with socially marginalized, low-income, and/or underhoused patients who may have trouble accessing or retaining the requisite communication technologies: *“[these patients] don’t have reliable ways for us to contact them and many of them don’t have phones*. *If they do have phones*, *half the time there was no minutes on their phone or no voicemail set up*…*”* [Participant D]. Additionally, for participants working with refugees, newcomers to Canada, and those for whom English is not their first or preferred language, virtual care could pose an added barrier to providing quality, patient-centred care: “*virtual care becomes another level of difficulty for people who don’t speak English well*, *who may not have access to enough bandwidth to support some of the options”* [Participant O]. In these instances, FPs noted the value of video over phone, even when working with an interpreter, to capture patients’ body language and physical gestures but which requires sufficient connectivity and suitable devices.

For individuals with access to technology but limited technological literacy, aspects of a virtual appointment posed barriers and participants recognized that it was not a suitable modality:

*…my patient population*, *the majority of them would not be able*, *even now*, *to login to certain sites and upload a visit*. *I think that if [virtual care] stays around*, *you’ll get the 20s-60s probably picking up on it in the future but the elderly population I don’t think will*. [Participant J]

And while virtual care has the potential to improve access for some patients in rural and remote areas by eliminating travel for in-person visits, this requires both patients and providers have adequate access to cellular or broadband service which not all communities have:

*I’ve got people in my community that don’t even have cell service where they are*. *So I think not that we get rid of it*, *but be very mindful that technology has allowed us to do a lot more but it’s not necessarily the panacea for everybody*. *That it has challenges in rural communities*. *I have lots of patients that don’t have cell service or internet service*. [Participant Q]

#### Quality and efficacy of care

Participants were surprised to realize that virtual care could be an equal or superior modality to in-person visits for some indications, facilitating the delivery of high quality care. For patients with more complex case management needs, participants suggested that virtual modalities contributed to improved quality of care through better management of lab testing, health counselling, and more frequent check-ins:

*For patients with diabetes […] Like I could call them and say*, *“oh*, *your sugars were 17 in the last four days*. *Okay*, *go up [on your medication] by two units*. *And I’m going to call you again next week”*. *You don’t have time to bring patients like that in once a week*. *[…] So it really creates an easy way to keep people out of the emergency room*, *and keep a better eye on patients with chronic diseases*. [Participant U]

There were, however, limitations to the type of care that FPs were confident providing virtually without compromising on quality. In this regard, the perceived value of virtual care varied by participant. Despite some participants’ perception that virtual care could help provide improved case management for patients, other participants felt that virtual was best reserved for more straightforward visits (such as for medication management or review of lab results):

*The vast majority of my virtual care visits were with folks that needed relatively simple things*. *And the vast majority of my in-person visits tended to be either acute injuries or folks with complex care needs*. *Because I just came to realize that you can’t really do that effectively over the phone*. [Participant L]

In part, this reflects an inherent limitation of virtual care–that the ability to perform a physical exam is limited. When using virtual care for more complex issues, one FP noted how much more time their consultation took due to the complexity and *“because you’re missing a lot of data that you can get in person much more quickly”* [Participant N]. While several FPs expressed an initial excitement about the introduction of virtual care, many quickly came to recognize its limitations:

*I also recognize that I have a far more fruitful*, *far more engaging*, *far more insightful conversation when someone’s in front of me and I can see them and I can touch them and I can examine them*, *and I can understand what’s going on*. [Participant Q]

Part of this can be attributed to the deficiencies of patients’ descriptions of their own symptoms, which can ultimately be misleading for FPs during virtual assessments: *“what nobody really takes into account is that when you come see your family physician*, *I’m using my own training to assess my description of what you have that may not be the same as what you describe it as”* [Participant B].

Participants’ concerns about the quality and appropriateness of virtual modalities frequently reflected their patients’ capacity. Dementia care in general and cognitive testing in particular were raised as examples of services that are difficult for FPs to do *“in a fair way [with] a telemedicine unready person”* [Participant A]. Another participant elaborated on their experiences attempting virtual care with this population:

*…these people have dementia so they’re struggling to understand why we’re talking to them on the phone*, *or you know*, *“Who is this person I can see on the computer screen here*?*” and then having to be reminded*, *it’s the doctor*. [Participant R]

Some of the participants’ concerns about the quality of virtual care were less about virtual care itself and more about managing patients’ preferences and ensuring they were still seeing patients in-person occasionally. This concern reflected FPs’ recognition that providing quality care via virtual modalities requires a hybrid approach wherein in-person visits are interspersed between virtual visits: “*there’s a lot of people who just think that telephone is more convenient or they don’t want to come in*. *And we’re trying to balance that in a way that’s best for patient safety”* [Participant M]. In these scenarios, FPs relied on existing relationships with patients to negotiate a compromise:

*I did have to politely fuss at some patients and be like*, *“no*, *you still have to come in for your foot exam*.*” Like*, *“just because there’s virtual care*, *it doesn’t mean that you never have to see me again*.*” I mean that’s just having conversations with your individual patients and knowing their personalities*. [Participant U]

### Comfort with virtual care

#### Patients

While FPs’ use of virtual care varied by patient and the reason for their appointment, participants perceived that virtual modalities were welcomed by many of their patients. Several participants commented on patients being happy about not needing to take time away from work, arrange childcare, or drive long distances for an in-person appointment:

*The opportunity cost of a medical visit is very high*. *You have to take the afternoon off work*. *And your doctor is always late*, *and this and that*. *And instead*, *you could schedule in a time*. *We would tell patients that [the FP] is going to be calling you within this hour period*. *And people who were working would just excuse themselves from work for 5 or 10 or 15 minutes*, *and then they’d be back at it*. *So the impact in terms of their professional and personal lives were very much less*. [Participant L]

Some FPs in the study expressed that the comfort of virtual care that they perceived from their patients was centred in the ease of connecting with their physician by video or phone:

*It’s become a joke in our clinic that actually*, *people are very comfortable with virtual visits and sometimes we’ll be doing video visits with people—the funniest one we’ve had is someone in the shower*, *they literally were like*, *“No*, *no*, *no*, *it’s fine*, *just keep talking*, *I’m in the shower*, *I’ll just set the phone up here*,*” and just… Anyway*, *people are comfortable*. [Participant O]

Other participants expressed how that patient comfort could have therapeutic benefits. This was specifically highlighted by an FP working in a primary care eating disorder clinic:

*Some of them feel more comfortable in their home environment*, *where they don’t have to show their entire bodies and they don’t get triggered by looking at other people’s bodies*. *[…] they feel more safe in their own environment with their support animals and everything*, *so it’s kind of interesting*. [Participant T]

Conversely, other patients have expressed a lack of comfort for virtual care and FPs have adjusted accordingly. As one participant noted, “*I didn’t do video; I tried and the patients seemed to absolutely hate it*. *Nobody wanted that*. *So*, *after a while*, *I stopped offering it*” [Participant I]. Similarly, patient preference prompted participants to vary their approaches with virtual care to be more patient-centred:

*I understood from some patients*, *that they weren’t happy with phone visits*. *They’d much prefer in-person visits*. *Not all patients*, *some people love phone visits*, *some people think it’s the best*. *[…] I’ve learned that sometimes on a phone visit*, *it’s not natural sometimes*, *we have to get better*, *but some patients do not feel completely cared for*. *They don’t have that personal connection and so you have to kind of make an extra effort over the phone*. [Participant C]

#### Providers

Most participants themselves were comfortable using virtual modalities to provide care, especially once they had determined which types of care they could provide virtually and which required an in-person appointment. This comfort with virtual care was greatest amongst those participants who had prior experience with virtual modalities: *“it’s something I did before and it’s something I’m quite used to*. *[…] I don’t think there’s a difference between an in-office visit with me and a phone call visit with me”* [Participant P]. For those with limited or no virtual care experience prior to COVID-19, comfort was often a product of acquiring experience over the course of the pandemic.

Where participants expressed discomfort with virtual care was in relation to their patients’ level of engagement in virtual visits. Several participants noted their frustration around patients multitasking during or not being sufficiently prepared for their virtual appointments:

And patients are sometimes not prepared to actually have an appointment when you call them.[…] They’re not coming to the office to talk about the problem, so they don’t necessarily have the information that they would normally have collected together to come in. Sometimes they’re in their car, they’re shopping. It doesn’t feel like an appointment, so sometimes they’re not as prepared. [Participant N]This may reflect a broader concern that virtual care had altered patients’ expectations and perceptions of primary care. Participants commented that the availability of phone visits meant that patients expected they could reach their physician at any time: *“there would be a lot of people calling up and wanting to talk to us like*, *right away*, *which was kind of complicated*” [Participant M]. Sometimes, this resulted in patients expressing their frustration and being verbally aggressive toward FPs’ clerical staff. In other cases, FPs noticed that their use of virtual care could lead some patients to infer their clinic was closed and that, by extension, telephone or video visits did not count as “work”:

*They wouldn’t call*. *They would just tell the pharmacy*, *“My doctor’s closed*. *Can you get my prescriptions refilled by fax*?*” … Sometimes people still say*, *“Oh*, *when are you going to re-open*?*” I’m literally talking to you on the phone*. [Participant N]

### Supports for continued use of virtual care

Many participants spoke strongly in favour of continuing to offer virtual care to support their patients as part of routine primary care. In response to an interview question about what pandemic changes should be retained beyond the pandemic recovery phase, one participant’s response was emphatic: *“Phone*, *phone*, *phone*, *phone*, *phone*, *phone*, *phone*, *phone*, *phone”* [Participant H].

FPs in the study explained how they had, through their experiences implementing virtual care during the pandemic, become comfortable relying on their clinical judgement and existing relationships with patients to determine the appropriateness of virtual care. Still, several participants expressed an interest in practice guidelines to support those decisions: *“it would be nice to have more deliberate strategy around how often someone should be seen in-person*, *which I don’t think we have very good systems for yet*. *Like*, *every second prescription at least should be done in-person for diabetics”* [Participant M].

To support ongoing and equitable access to virtual care, FPs spoke about expanding supports for patients who might not have access to or comfort with technology. For example, to improve remote access for underhoused patients, physicians recommended partnerships with community organizations who could provide safe spaces and the requisite technology to navigate virtual care:

*So*, *in our [low income housing]*, *could there be an iPad set up*? *Could there be a little room with one of those ring lights and an iPad set up so that*, *“Okay*, *Samantha*, *you’ve got your appointment with your doctor at 10*:*00 and so you’re going to have that room for that half an hour*, *so that you can have your Zoom appointment with your doctor*.*”* [Participant E]

Likewise, another FP recommended facilities akin to internet cafés “where people could access virtual health if they didn’t have access to their own [technologies]” [Participant K].

Even for patients with access to the requisite phone or video devices and adequate connectivity, some care requires medical devices. For example, to facilitate routine virtual care for diabetic patients, multiple FPs recommended that patients purchase or be supplied with blood pressure monitors:

*For some things we do repetitively over and over again–diabetic checks and hypertensive checks–they were a little bit easier*. *I taught my patients to go and buy themselves a blood pressure cuff and then to either fax in or call in ahead of time those pressures so we could talk about whether we need to make any changes*. [Participant G]

## Discussion

The rapid introduction of virtual care driven by the COVID-19 pandemic facilitated ongoing access to care during lockdowns or periods where in-person visits posed a high risk to clinicians and patients [1, 2]. This study adds to a growing body of literature that examines physicians’ perspectives on the use of virtual care to support their patient populations during a time of restricted in-person access [41–43], highlighting the variability in the implementation and use of virtual modalities across individual physicians and patient groups during the first year-and-a-half of the pandemic in Canada. Data from our interviews demonstrated the various ways FPs leveraged their existing relationships with patients, and their clinical skills, to guide their decisions on which patients could be seen virtually versus which patients needed to come to the clinic for an in-person visit. While we focus here primarily on physician perspectives of the strengths and challenges of virtual modalities for their specific patient populations, we recognize the impact of context, such as provincial and intra-provincial remuneration policies and practice models–on physician decisions to opt for one care modality over another. We explore these additional considerations in other related work [2].

Physicians spoke about the ways virtual care could enhance access to care for some of their patients, improving timeliness as well as the comfort and convenience of medical visits. Consistent with existing research, our FP interviews highlighted the ways that virtual visits conducted from the comfort of patients’ homes could reduce their time away from work, or the need to arrange childcare or transportation [44]. Physicians also noted the ways in which virtual care enhanced their ability to have more frequent touchpoints with patients, with the potential for improving mental health care [45–47], chronic disease management [47–52], and preventative care [53] for some patients. These more frequent visits could also strengthen physician-patient relationships and improve continuity of care [54, 55].

At the same time, the wholesale move to virtual care created challenges for other patient groups and for some types of care, exposing the limitations of this modality. The physicians we interviewed expressed concerns that their elderly patients, many of whom had limited technological literacy, struggled with virtual care. This aligns with existing research demonstrating that older adults are less likely to engage in virtual video visits compared with younger adults [56], although evidence indicates similar or better health outcomes from virtual care amongst older adults compared to in-person appointments [57]. They also noted that, while virtual care was particularly useful for patients who lived in rural communities who had to travel long distances to seek care, these patients also had less ability to benefit from virtual access due to poorer internet and telephone services where they lived [58, 59]. Reliance on virtual care can also exacerbate existing access inequities among low-income communities [20, 60], and many FPs we spoke with provided examples of why telephone or video consultations simply did not work for their underhoused or socially marginalized patients–even when those patients would have liked access to a virtual appointment [61].

While the physicians we spoke with often mentioned the need for evidence-informed guidelines to assist decision-making around which patients to see in-person and when, decisions about visit modalities must be centred on the needs and preferences of patients, and physicians’ clinical judgement [13, 62]. Discussing their experiences of providing virtual care in the context of their longitudinal family practices, our participants identified positive uses of virtual care in established, ongoing relationships with their patients, where they had a good understanding of patients’ conditions, and social and family circumstances. These ongoing relationship and patient-centred approaches are integral to high quality care [20, 63–65], and act as a counter to the potential negative impacts of virtual care on continuity and fragmentation of care when used in an episodic, walk-in style, as discussed elsewhere [5, 66].

The effective integration of virtual modalities into primary care practice will necessitate an evolution of roles and responsibilities for both patients and clinicians. For example, FPs may need to articulate their expectations for patients in terms of appropriate settings for visits and required preparation for appointments (e.g., having access to current prescriptions, collecting and recording of blood pressure). For patients and for policy makers, this may require broader investments in infrastructure to support access to care–including access to internet, provision of community spaces to access virtual visits, and the provision of personal medical devices for self-management and monitoring. These investments are necessary to ensure equitable integration of virtual visits into routine primary care.

### Limitations

Data collection for this study took place in four regions across Canada in the early phases of the pandemic between October 2020 and June 2021. While the regions were selected due to variability in terms of their pandemic experiences and responses as well as varieties of primary care practice models, our results may not be applicable to other jurisdictions or later phases of the pandemic (when the balance of virtual to in-person care had shifted back to more in-person visits from majority virtual visits, and when both clinicians and patients had had more experience with using virtual care [1, 67]). Though we used maximum variation sampling and varied recruitment strategies, our sample may not be representative of the demographic composition and practice characteristics of FPs in Canada. In particular, we note the disproportionate representation of women, rural physicians, and those compensated by alternative payment plans in our sample, relative to national averages in Canada [68, 69]. Additionally, we did not ask our participants any direct questions about their experiences with or perspectives on virtual care; however, comments on virtual care arose organically in all interviews given the salience of the transition to virtual care that FPs experienced in the early phase of the pandemic. Comments specifically addressed the use of synchronous telephone and video visits. Participants did not comment on the use asynchronous forms of virtual care such as email or chat. As our interviews were conducted exclusively with FPs, we are not able to provide first-hand perspectives on patient experiences and preferences; future research should be conducted with patients directly to build on the FP portrayals that we have included here. Finally, interviews are subject to recall and response biases [70, 71].

## Conclusions

FPs who integrated virtual care into their practices early in the pandemic reported that virtual care improved access to care for many of their patients, but created challenges for others. Evidence-informed guidance, regulation, and investments in infrastructure, are needed to support the ongoing integration of virtual care into broader primary care delivery in a way that ensures equitable access and maximizes quality of care for all patients.

## Supporting information

S1 File Interview guide. (DOCX)
